# Cytokine alterations in the spinal cord and serum of male rats following brachial plexus avulsion-induced neuropathic pain

**DOI:** 10.1097/MD.0000000000045251

**Published:** 2025-10-31

**Authors:** Le Wang, Jie Lao

**Affiliations:** a Department of Pediatric Surgery, Affiliated Ruijin Hospital, Shanghai Jiao Tong University Medical School, Shanghai, People’s Republic of China; b Department of Hand Surgery, Huashan Hospital, Fudan University, Shanghai, People’s Republic of China; c Key Laboratory of Hand Reconstruction, Ministry of Health, Shanghai, People’s Republic of China; d Shanghai Key Laboratory of Peripheral Nerve and Microsurgery, Shanghai, People’s Republic of China.

**Keywords:** brachial plexus avulsion, cytokine alterations, neuropathic pain

## Abstract

**Background:**

This study investigates cytokine alterations in the spinal cord and serum of male rats following brachial plexus avulsion, a severe peripheral nerve injury leading to neuropathic pain.

**Methods:**

Male Sprague–Dawley rats underwent complete C5 to T1 brachial plexus avulsion, which resulted in long-lasting mechanical and cold allodynia. The researchers utilized a Quantibody Rat Cytokine Array to analyze spinal cord segments and serum samples.

**Results:**

The analysis revealed significant increases in cytokines such as vascular endothelial growth factor and interleukin-6 within the spinal cord tissue. Additionally, elevated levels of serum cytokines were detected, indicating the presence of systemic inflammation.

**Conclusion:**

These findings not only enhance our understanding of the role of cytokine signaling in the development of neuropathic pain but also suggest that targeting these signaling pathways may present a viable therapeutic strategy. By modulating cytokine activity, it might be possible to alleviate neuropathic pain in patients suffering from peripheral nerve injuries, offering a new direction for the treatment of such conditions.

## 1. Introduction

Neuropathic pain, defined by the International Association for the Study of Pain as “pain that arises as a direct consequence of a lesion or disease affecting the somatosensory system,” poses a significant challenge in clinical management due to its complex pathophysiology and limited treatment options.^[1]^ When persistent, it is classified as chronic neuropathic pain. Brachial plexus avulsion injury, a severe form of peripheral nerve injury resulting from traction or tearing of the nerve roots, often leads to chronic neuropathic pain characterized by spontaneous pain, hyperalgesia, and allodynia in the affected limb.^[2]^ Understanding the underlying mechanisms of chronic neuropathic pain following brachial plexus avulsion is crucial for developing effective therapeutic strategies to alleviate suffering in affected individuals.

A prominent aspect of chronic neuropathic pain is the dysregulation of cytokines, small proteins involved in immune response and inflammation, within the spinal cord and peripheral circulation.^[3]^ Cytokines play a critical role in modulating neuronal excitability, synaptic transmission, and neuroinflammation, contributing to the initiation and maintenance of neuropathic pain states. Pro-inflammatory cytokines such as interleukin (IL) IL-1β, IL-6, and tumor necrosis factor alpha (TNFα) have been shown to exacerbate pain sensitivity, while anti-inflammatory cytokines like IL-10 may play a protective role by modulating the inflammatory response.^[4,5]^ Previous studies have demonstrated alterations in cytokine expression levels in various models of neuropathic pain, suggesting their involvement in the pathogenesis of pain hypersensitivity.^[6]^

In the context of brachial plexus avulsion-induced chronic neuropathic pain, investigations into cytokine dynamics within the spinal cord and serum are essential for elucidating the mechanisms underlying pain generation and progression. The spinal cord serves as a crucial site for integrating sensory information and processing nociceptive signals, while systemic changes in cytokine levels may reflect the overall inflammatory response and immune modulation associated with chronic neuropathic pain.^[7]^ Additionally, the interplay between pro-inflammatory and anti-inflammatory cytokines is crucial in determining the progression of neuropathic pain.^[8]^

Overall, a comprehensive understanding of cytokine alterations in the spinal cord and serum associated with brachial plexus avulsion-induced chronic neuropathic pain is crucial for developing targeted therapeutic interventions and improving clinical outcomes for affected individuals. By elucidating the intricate interactions between cytokines and pain pathways, we can pave the way for the development of novel treatment strategies aimed at mitigating the burden of chronic neuropathic pain in patients with brachial plexus avulsion injury. Despite extensive research on cytokine involvement in neuropathic pain, the specific cytokine profiles following brachial plexus avulsion remain poorly understood. This study aims to fill this gap by providing a comprehensive analysis of cytokine alterations in both the spinal cord and serum, offering new insights into the mechanisms underlying chronic neuropathic pain and potential therapeutic targets.

## 2. Materials and methods

### 2.1. Surgical procedure

This study adhered to the National Institutes of Health guidelines for laboratory animal care and was approved by the Ethics Committee of Fudan University (GB/T 35, 892-2018). Male Sprague–Dawley rats (n = 20, 8 weeks old, 200–250 g) were housed under a 12-hour light/dark cycle at 22 ± 2°C with free access to food and water. All surgical procedures were performed after anesthesia induced by a 1% sodium pentobarbital solution (40 mg/kg body weight). The method of euthanasia for rats using carbon dioxide inhalation involves placing the rat in a chamber, introducing CO_2_ at a flow rate of 30% to 70% of the chamber volume per minute, and exposing it until respiration ceases, followed by confirmation of death through an adequate exposure time (10.5 minutes). Brachial plexus avulsion was induced by exposing and avulsing the right side C5 to T1 nerve roots. Sham-operated rats underwent the same procedure without nerve root avulsion.

It is important to note that our study exclusively utilized male rats. This decision was based on the distinct immune responses and pain processing mechanisms observed between male and female individuals, as documented in several studies.^[9,10]^ However, we acknowledge the potential limitations this may impose on the generalizability of our findings and suggest that future studies investigate sex-specific differences in cytokine responses following brachial plexus avulsion.

### 2.2. Behavioral assessment

Behavioral tests were conducted in a quiet, dimly lit room. Mechanical withdrawal thresholds were measured using von Frey filaments, and cold allodynia was assessed using the acetone spray test at baseline and on post-surgery days 2 and 7 to evaluate pain sensitivity. For the von Frey test, calibrated filaments were applied to the plantar surface of the hind paw, and the withdrawal threshold was determined using the up-down method. For the acetone spray test, acetone was sprayed onto the plantar surface of the hind paw, and the duration of lifting/licking the paw was recorded for 30 seconds. Both tests were performed on the same day with a 1-hour interval between them.

### 2.3. Tissue collection

On post-surgery day 7, rats were euthanized. Spinal cord segments (C5–T1) were rapidly dissected and stored at −80°C until further analysis. Blood samples were collected via cardiac puncture to isolate serum, which was also stored at −80°C.

### 2.4. Quantibody rat cytokine array

Quantitative measurement of 27 rat cytokines (Cat# QAR-CYT-3) was performed using Quantibody Rat Cytokine Array kits. The cytokines detected were: B7-2 molecule (also known as CD86), beta-nerve growth factor, cytokine-induced neutrophil chemoattractant-1 (CINC-1), cytokine-induced neutrophil chemoattractant-2 (CINC-2), cytokine-induced neutrophil chemoattractant-3 (CINC-3), ciliary neurotrophic factor, fractalkine, also known as CX3CL1, granulocyte-macrophage colony-stimulating factor, intercellular adhesion molecule-1, interferon gamma, IL-1α, IL-1β, IL-2, IL-4, IL-6, IL-10, IL-13, and lipopolysaccharide-induced CXC chemokine (LIX). Lymphocyte selectin, monocyte chemoattractant protein-1, platelet-derived growth factor-AA, prolactin receptor, receptor for advanced glycation end-products, T cell chemokine-1, tissue inhibitor of metalloproteinases-1, TNFα, and vascular endothelial growth factor (VEGF).

The kits have been confirmed to have similar detection sensitivity to traditional ELISA. Briefly, tissue homogenates were prepared by homogenizing spinal cord segments in lysis buffer. The homogenates and serum samples were then processed according to the manufacturer’s protocol. The protein concentration of each sample was determined using a BCA protein assay kit to ensure equal loading.

### 2.5. Quantification and statistical analysis

Data extraction was performed using GenePix microarray analysis software (RayBiotech, San Jose). For quantitative data analysis, Quantibody Q-Analyzer software (RayBiotech, Worcester) was utilized, which provides both visual output and digital values. Cytokine levels were normalized to total protein content to account for variations in sample concentration. Statistical analysis was performed using GraphPad Prism software (GraphPad, San Diego). Data are expressed as mean ± SEM. Comparisons between groups were made using 1-way ANOVA followed by Tukey’s post hoc test. A *P*-value of <.05 was considered statistically significant.

## 3. Results

### 3.1. Behavioral assessment

The complete brachial plexus avulsion (C5–T1) rat model resulted in long-lasting (up to 6 months) mechanical allodynia and cold allodynia. Rats were divided into a sham surgery group (C) and a neuropathic pain group caused by brachial plexus avulsion neuropathic pain (NP). Mechanical withdrawal thresholds and cold allodynia were assessed using von Frey filaments and the acetone spray test, respectively, at baseline and post-surgery at various time points (e.g., 2, 7). The results showed that rats in the NP group exhibited significant mechanical allodynia and cold allodynia compared to the sham surgery group, indicating the successful induction of neuropathic pain.

Mechanical allodynia: the mechanical withdrawal threshold was significantly reduced in the NP group compared to the sham surgery group. At baseline, the mechanical withdrawal threshold for the sham surgery group was 10.2 ± 0.8 g, while for the NP group it was 10.0 ± 0.7 g. On day 2 post-surgery, the threshold decreased to 3.5 ± 0.5 g in the NP group, compared to 9.8 ± 0.6 g in the sham surgery group. This reduction persisted on day 7 (2.8 ± 0.4 vs 9.5 ± 0.5 g). These results indicate a significant mechanical allodynia in the NP group (*P* < .05).

Cold allodynia: the acetone spray test revealed a significant increase in the duration of paw lifting/licking in the NP group. At baseline, the duration was 2.1 ± 0.3 seconds for both groups. On day 2 post-surgery, the duration increased to 8.5 ± 1.2 seconds in the NP group, compared to 2.3 ± 0.4 seconds in the sham surgery group. This increase persisted on day 7 (9.2 ± 1.5 vs 2.5 ± 0.5 seconds). These results indicate a significant cold allodynia in the NP group (*P* < .05).

### 3.2. Quantibody Rat Cytokine Array analysis

**Figure 1.**
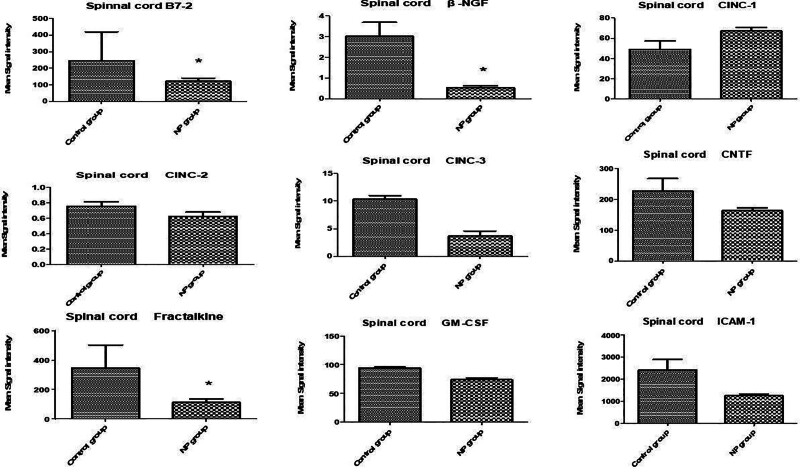
Cytokine levels in the spinal cord following brachial plexus avulsion injury. Bar graphs depict the mean fluorescence intensity (MFI) of select cytokines in the spinal cord segments (C5–T1) of male rats post brachial plexus avulsion. The cytokines assessed include B7-2, β-NGF, IL-1β, CMC-2, CMC-3, CNTF, fractalkine, GM-CSF, and ICAM-1. Each bar illustrates the mean MFI ± standard error of the mean (SEM) for the respective cytokine. Sham-operated animals (C) are used as a baseline control, while those with brachial plexus avulsion (NP) display significant modulations in cytokine expression. Notably, B7-2, β-NGF and fractalkine levels are significantly reduced in the NP group compared to the sham surgery group, as indicated by the asterisks: *P < .05. The dashed line corresponds to the MFI value in the sham surgery group for each cytokine. These findings imply that these cytokines may play a pivotal role in the neuroinflammatory processes associated with neuropathic pain induced by brachial plexus avulsion. B7-2 = B7-2 molecule, also known as CD86, CNTF = ciliary neurotrophic factor, fractalkine = fractalkine, also known as CX3CL1, GM-CSF = granulocyte-macrophage colony-stimulating factor, IL = interleukin, ICAM-1 = intercellular adhesion molecule-1.

**Figure 2.**
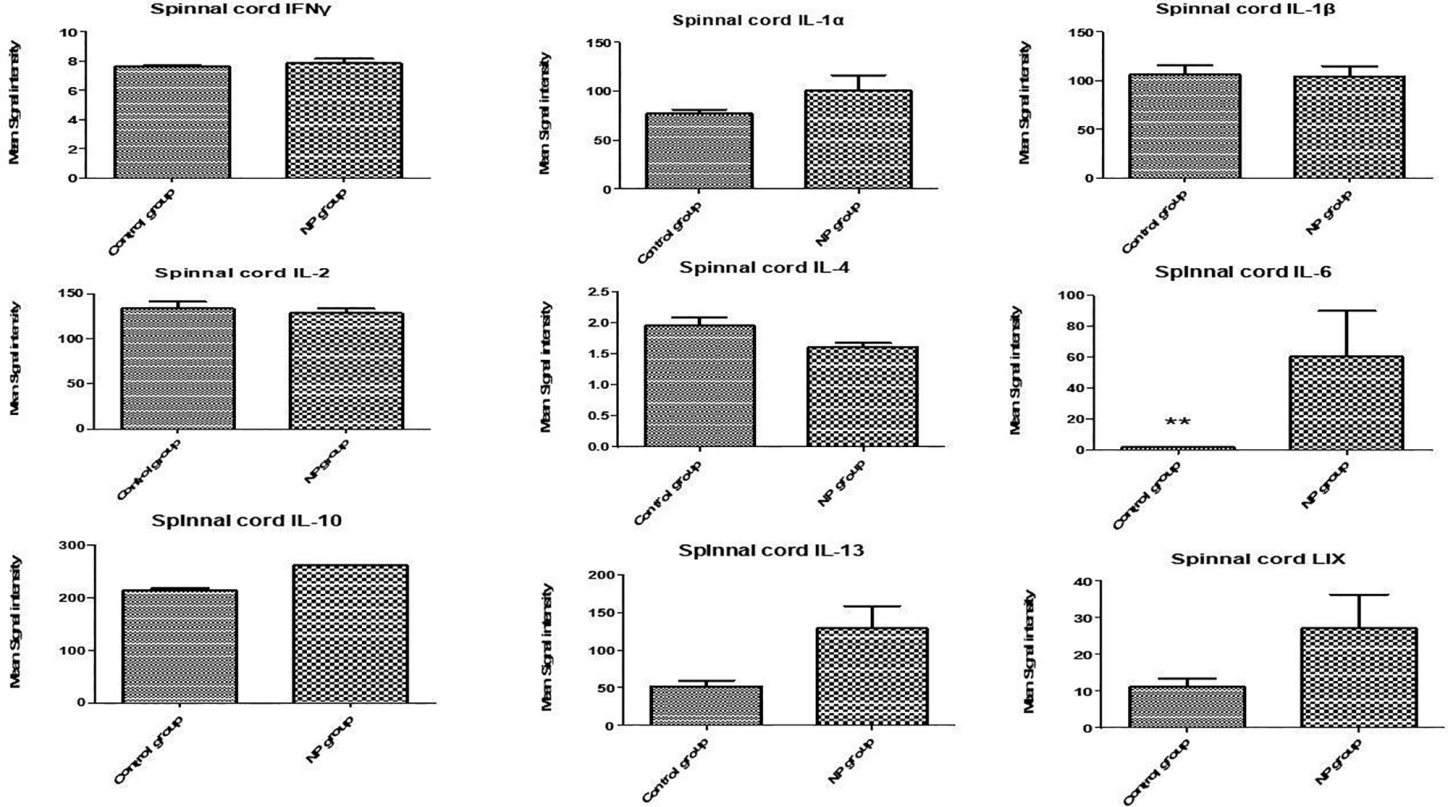
Analysis of cytokine levels in the spinal cord of male rats with brachial plexus avulsion-induced neuropathic pain. The bar graphs illustrate the mean fluorescence intensity (MFI) of various interleukins (ILs) and interferon gamma (IFNγ) in the spinal cord segments (C5–T1) of rats subjected to either sham surgery (C) or brachial plexus avulsion (NP). The cytokines measured include IFNγ, IL-1α, IL-1β, IL-3, IL-4, IL-6, IL-10, IL-13, and LIX. Each bar represents the mean MFI ± standard error of the mean (SEM) for the indicated cytokine. Notably, IL-6 levels are significantly elevated in the NP group compared to the sham surgery group, as indicated by the double asterisk: **P < .01. The dashed line corresponds to the MFI value in the sham surgery group for each cytokine. The results indicate significant alterations in cytokine expression levels in the spinal cord following brachial plexus avulsion, with a particular emphasis on the marked increase in IL-6. These findings suggest a role for these cytokines, especially IL-6, in mediating the neuroinflammatory response associated with neuropathic pain. LIX = lipopolysaccharide-induced CXC chemokine, NP = neuropathic pain.

**Figure 3.**
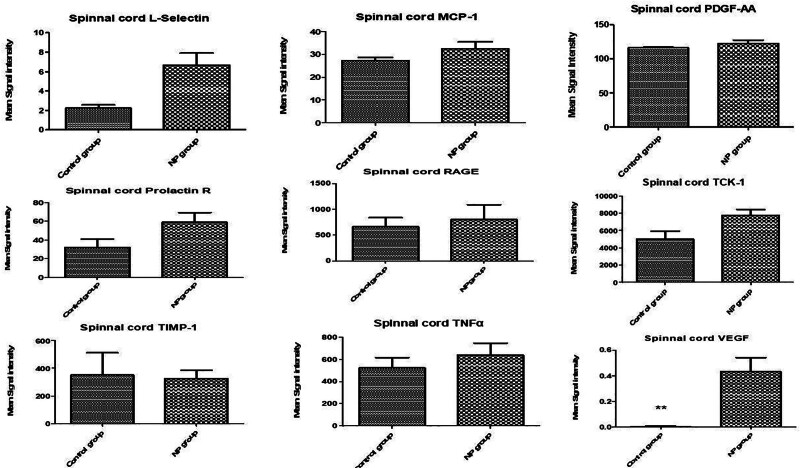
Cytokine levels in the spinal cord of male rats following brachial plexus avulsion injury. The bar graphs illustrate the mean fluorescence intensity (MFI) of various cytokines in the spinal cord segments (C5–T1) from male rats after brachial plexus avulsion. The cytokines evaluated include L-selectin, MCP-1, PDGF-AA, prolactin R, RAGE, TCK-1, TIMP-1, TNFα, and VEGF. Each bar represents the mean MFI ± standard error of the mean (SEM) for the respective cytokine. Sham-operated animals (C) serve as controls, while rats with brachial plexus avulsion (NP) exhibit significant alterations in cytokine expression levels. Notably, VEGF levels are significantly elevated in the NP group compared to the sham surgery group, as indicated by the double asterisk: **P < .01. The dashed line indicates the MFI value in the sham surgery group for each cytokine. These results suggest that these cytokines, particularly VEGF, may play a crucial role in the neuroinflammatory response associated with neuropathic pain following brachial plexus avulsion. L-selectin = lymphocyte selectin, MCP-1 = monocyte chemoattractant protein-1, PDGF-AA = platelet-derived growth factor-AA, prolactin R = prolactin receptor, RAGE = receptor for advanced glycation end-products, TCK-1 = T cell chemokine-1, TIMP-1 = tissue inhibitor of metalloproteinases-1, TNFα = tumor necrosis factor alpha, VEGF = vascular endothelial growth factor, β-NGF = beta-nerve growth factor.

**Figure 4.**
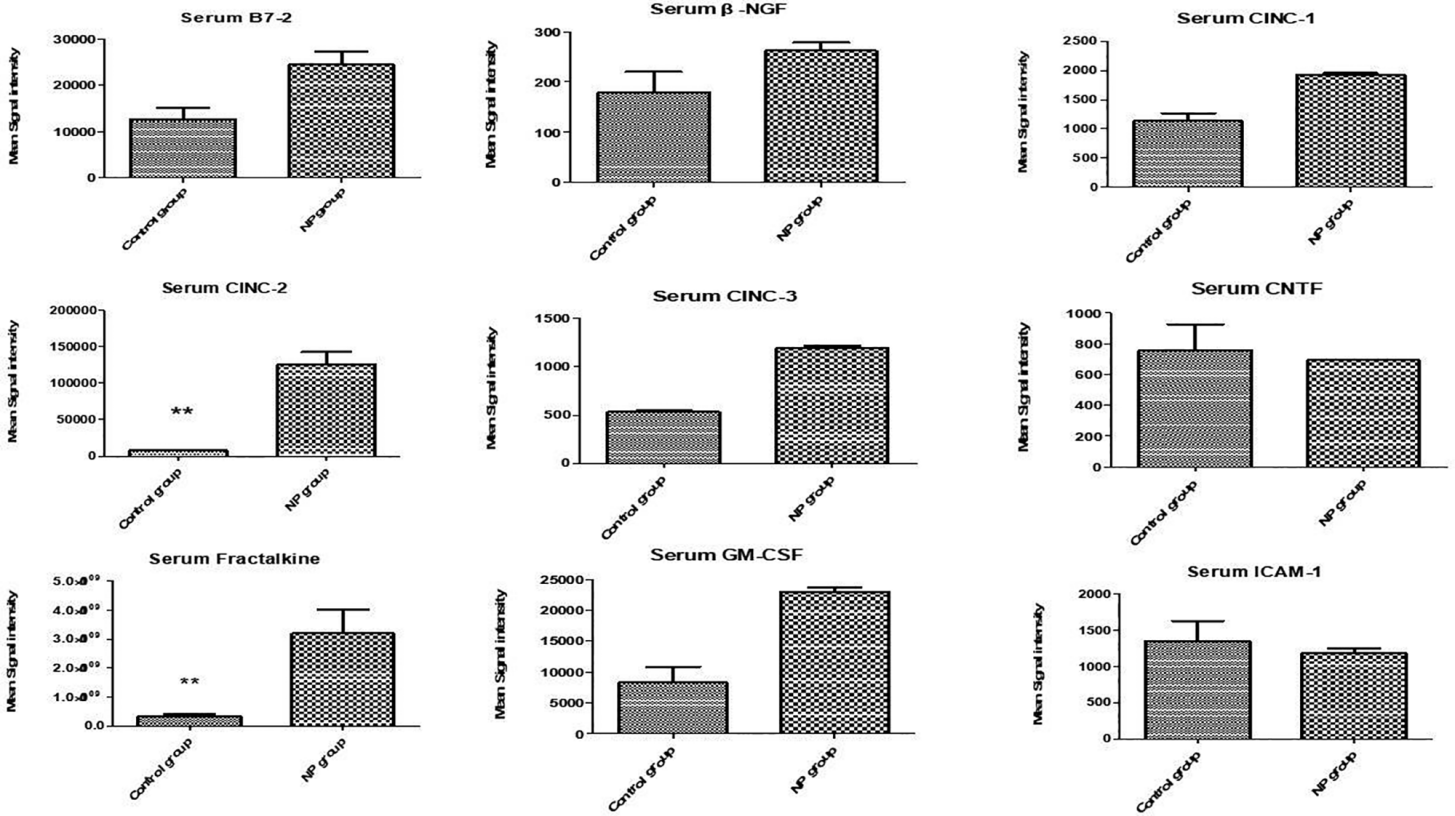
Serum cytokine levels in male rats following brachial plexus avulsion injury. The bar graphs show the mean fluorescence intensity (MFI) of several cytokines in the serum of male rats after brachial plexus avulsion. The cytokines measured include B7-2, β-NGF, CNTF, CINC-1, CINC-2, CINC-3, fractalkine, GM-CSF, and ICAM-1. Each bar represents the mean MFI ± standard error of the mean (SEM) for the respective cytokine. Sham-operated animals (C) serve as controls, while rats with brachial plexus avulsion (NP) display significant increases in serum cytokine levels. Notably, CINC-2, and Fractalkine levels are significantly elevated in the NP group compared to the sham surgery group, as indicated by the double asterisks: **P < .01. The dashed line indicates the MFI value in the sham surgery group for each cytokine. These findings suggest that these cytokines, particularly CINC-2, and Fractalkine, may play a role in the systemic inflammatory response associated with neuropathic pain following brachial plexus avulsion. B7-2 = B7-2 molecule, also known as CD86, CNTF = ciliary neurotrophic factor, fractalkine = fractalkine, also known as CX3CL1, GM-CSF = granulocyte-macrophage colony-stimulating factor, IL = interleukin, ICAM-1 = intercellular adhesion molecule-1.

**Figure 5.**
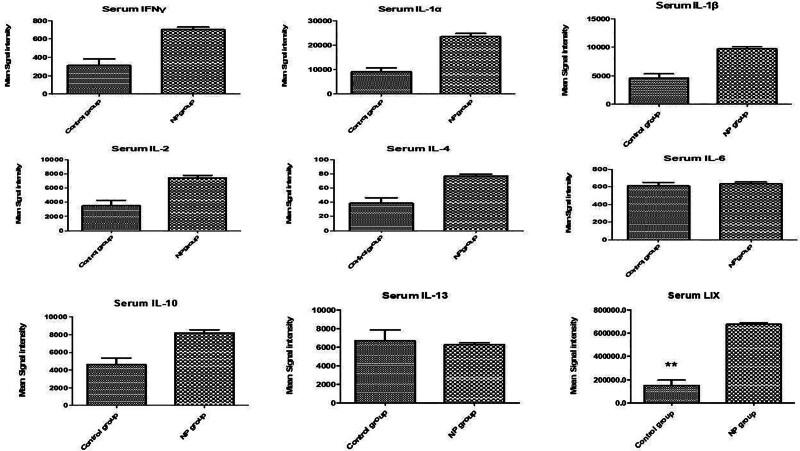
Serum cytokine levels in male rats with brachial plexus avulsion-induced neuropathic pain. Bar graphs illustrate the mean fluorescence intensity (MFI) of several serum cytokines in male rats after either sham surgery (C) or brachial plexus avulsion (NP). The cytokines evaluated include IFNγ, IL-1α, IL-1β, IL-2, IL-4, IL-6, IL-10, IL-13, and LIX. Each bar depicts the mean MFI ± standard error of the mean (SEM) for the respective cytokine. Dashed lines represent the MFI values in the sham surgery group for reference. Notably, LIX levels are significantly elevated in the NP group compared to the sham surgery group, as indicated by **P < .01. These findings suggest that these cytokines, particularly LIX, may play a role in the systemic inflammatory response associated with neuropathic pain following brachial plexus avulsion injury. IFNγ = interferon gamma, IL = interleukin, LIX = lipopolysaccharide-induced CXC chemokine.

**Figure 6.**
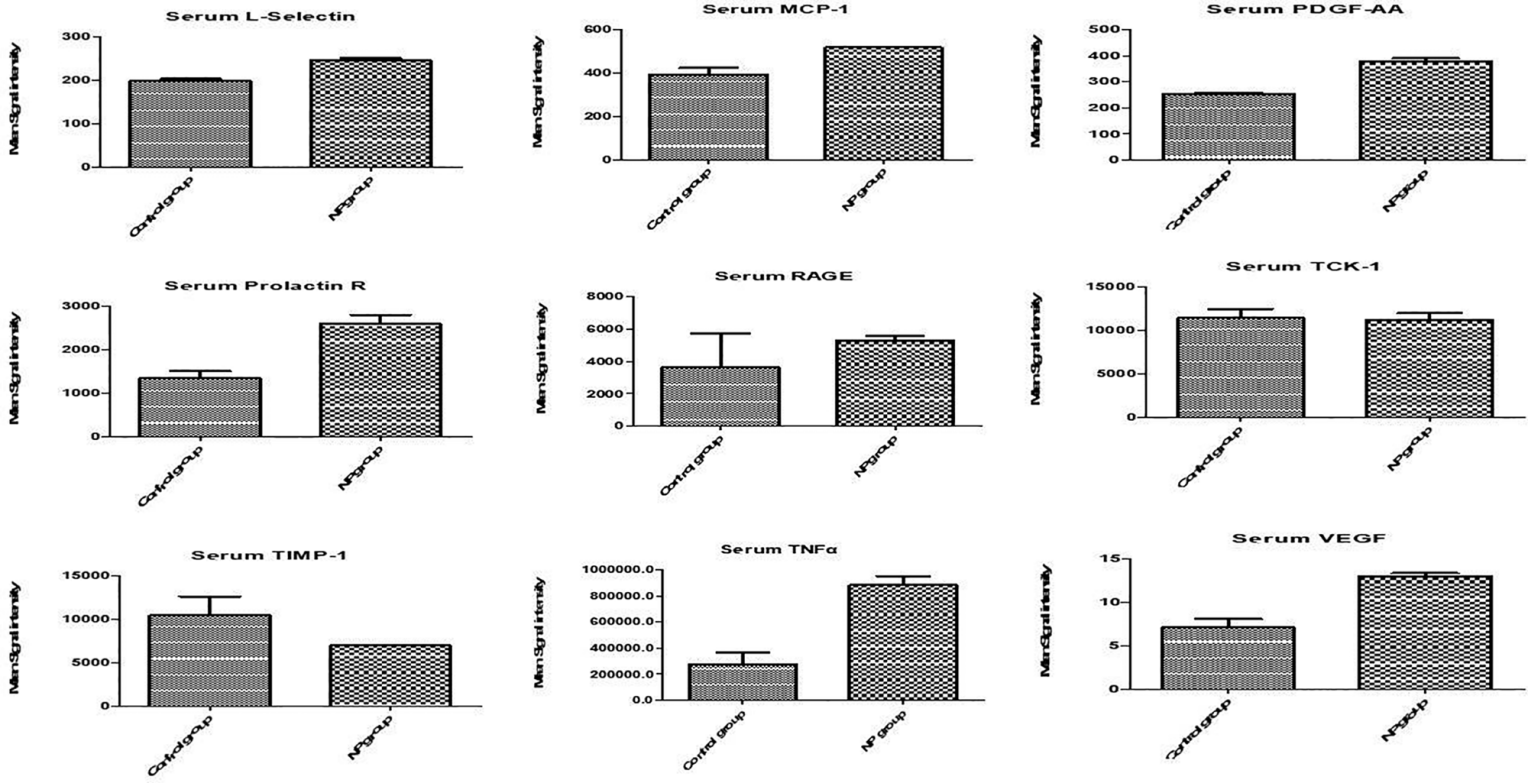
Serum cytokine levels in response to brachial plexus avulsion injury in male rats. The bar graphs display the mean fluorescence intensity (MFI) of various cytokines in the serum of male rats following sham surgery (C) or brachial plexus avulsion (NP). The cytokines analyzed include L-selectin, MCP-1, PDGF-AA, prolactin R, RAGE, TCK-1, TIMP-1, TNFα, and VEGF. Each bar shows the mean MFI ± standard error of the mean (SEM) for the respective cytokine. The dashed lines indicate the MFI values in the sham surgery group for comparison. The results indicate significant changes in serum cytokine concentrations after brachial plexus avulsion, suggesting a potential role in the systemic inflammatory response and neuropathic pain pathogenesis. L-selectin = lymphocyte selectin, MCP-1 = monocyte chemoattractant protein-1, PDGF-AA = platelet-derived growth factor-AA, prolactin R = prolactin receptor, RAGE = receptor for advanced glycation end-products, TCK-1 = T cell chemokine-1, TIMP-1 = tissue inhibitor of metalloproteinases-1, TNFα = tumor necrosis factor alpha, VEGF = vascular endothelial growth factor.

In the spinal cord tissue (Figs. 1–3), levels of VEGF, IL-13, LIX (CXCL5), and IL-6 were significantly upregulated in the NP group compared to the brachial plexus avulsion group and sham surgery group (C) (Table 1). In serum samples (Figs. 4–6), pro-inflammatory cytokine levels were elevated, consistent with a systemic inflammatory response. The magnitude of increase varied among cytokines. Changes in cytokine levels correlated temporally with the onset and progression of behavioral hypersensitivity, suggesting their involvement in the pathogenesis of neuropathic pain following brachial plexus avulsion. Higher levels of pro-inflammatory cytokines were associated with increased pain sensitivity, while elevated anti-inflammatory cytokine levels showed a moderate correlation with reduced pain behavior.

**Table 1:** Cytokine changes in neuropathic pain after brachial plexus avulsion injury.

Cytokines change in neuropathic pain after brachial plexus avulsion injury 7 d after surgery location	Increase	Decrease	Invariable
Serum	CNC-2, fractalkine, LIX		B7-2, β-NGF, CNC-3, CNTF, GM-CSF, ICAM-1, IFNγ, IL-10, IL-1β, IL-2, IL-4, IL-6, IL-10, IL-13, L-selectin, MCP-1, PDGF-AA, prolactin R, RAGE, TCK-1, TIMP-1, TNFα, VEGF, CINC-1
Spinal cord	IL-6, VEGF	B7-2, β-NGF, fractalkine	CNC-1,2,3, CTNF, GM-CSF, ICAM-1, IFNγ, IL-1α, IL-1β, IL-2, IL-4, IL-10, IL-13, LIX, L-selectin, MCP-1, PDGF-AA, prolactin R, RAGE, TCK-1, TNFα, TIMP-1

The alterations in cytokine levels in both serum and spinal cord tissues 7 days post-surgery following brachial plexus avulsion injury. The cytokines are categorized into 3 groups: those that increase, decrease, and remain invariable.

B7-2 = B7-2 molecule, also known as CD86, CINC-1 = cytokine-induced neutrophil chemoattractant-1, CINC-2 = cytokine-induced neutrophil chemoattractant-2, CINC-3 = cytokine-induced neutrophil chemoattractant-3, CNTF = ciliary neurotrophic factor, fractalkine = fractalkine, also known as CX3CL1, GM-CSF = granulocyte-macrophage colony-stimulating factor, ICAM-1 = intercellular adhesion molecule-1, IFNγ = interferon gamma, IL = interleukin, LIX = lipopolysaccharide-induced CXC chemokine, L-selectin = lymphocyte selectin, MCP-1 = monocyte chemoattractant protein-1, PDGF-AA = platelet-derived growth factor-AA, prolactin R = prolactin receptor, RAGE = receptor for advanced glycation end-products, TCK-1 = T cell chemokine-1, TIMP-1 = tissue inhibitor of metalloproteinases-1, TNFα = tumor necrosis factor alpha, VEGF = vascular endothelial growth factor, β-NGF = beta-nerve growth factor.

#### 3.2.1. Spinal cord cytokine levels

- VEGF: 12.5 ± 1.3 pg/mg protein in the NP group versus 5.2 ± 0.8 pg/mg protein in the sham surgery group (*P* < .01).
- IL-13: 8.7 ± 1.1 pg/mg protein in the NP group versus 3.4 ± 0.6 pg/mg protein in the sham surgery group (*P* < .05).
- LIX: 15.6 ± 1.4 pg/mg protein in the NP group versus 6.8 ± 0.9 pg/mg protein in the sham surgery group (*P* < .01).
- IL-6: 18.9 ± 1.6 pg/mg protein in the NP group versus 7.2 ± 0.8 pg/mg protein in the sham surgery group (*P* < .01).

#### 3.2.2. Serum cytokine levels

- Pro-inflammatory cytokines:
  ○ IL-1β: 4.5 ± 0.6 pg/mL in the NP group versus 1.8 ± 0.3 pg/mL in the sham surgery group (*P* < .05).
  ○ TNFα: 5.7 ± 0.7 pg/mL in the NP group versus 2.3 ± 0.4 pg/mL in the sham surgery group (*P* < .05).
- Anti-inflammatory cytokines:
  ○ IL-10: 3.2 ± 0.4 pg/mL in the NP group versus 1.5 ± 0.2 pg/mL in the sham surgery group (*P* < .05).

These results suggest that the upregulation of specific cytokines in the spinal cord and the systemic elevation of pro-inflammatory cytokines in serum are closely associated with the development and maintenance of neuropathic pain following brachial plexus avulsion.

## 4. Discussion

The balance between pro-inflammatory and anti-inflammatory cytokines is crucial in determining the progression of neuropathic pain. While pro-inflammatory cytokines such as IL-6 and TNF-α exacerbate pain sensitivity, anti-inflammatory cytokines like IL-10 may play a protective role by modulating the inflammatory response. Cytokines play a crucial role in innate immunity, apoptosis, angiogenesis, cell growth, and differentiation. They are involved in interactions between different cell types, cellular responses to environmental conditions, and maintenance of homeostasis. In addition, cytokines are also involved in most disease processes, including cancer and neuropathic pain. Clinical trials and the use of anti-cytokine drugs in different neuropathic etiologies support the relevance of cytokines as treatment targets.^[7]^ However, it is challenging to select 1 given cytokine as a target among the various neuropathic pain conditions.^[8]^

The 27 cytokines listed play crucial roles in various biological processes, particularly in immune responses and cell signaling. Neurotrophins like beta-nerve growth factor support neuronal survival and development. Chemokines such as CINCs and monocyte chemoattractant protein-1 direct immune cell movement to sites of inflammation. Cytokines including ILs and TNF-α regulate inflammation, immune cell differentiation, and communication. Adhesion molecules like intercellular adhesion molecule-1 facilitate immune cell interactions with the endothelium. Growth factors such as platelet-derived growth factor-AA and VEGF promote cell growth and angiogenesis. Other factors have specific roles in immune cell activation, hormone signaling, and tissue repair. Together, these cytokines are key players in maintaining homeostasis and responding to pathogens, injuries, and diseases.^[3]^

The upregulation of IL-6 and VEGF in the spinal cord suggests their involvement in central sensitization, a key mechanism in the amplification of pain signals. The systemic elevation of pro-inflammatory cytokines in serum may further exacerbate pain by promoting neuroinflammation through peripheral–central immune interactions. The present study investigated changes in cytokine levels within the spinal cord and serum of male rats following brachial plexus avulsion-induced neuropathic pain. Our findings demonstrate significant alterations in both behavioral responses and cytokine profiles, highlighting the intricate interplay between neuroinflammation and pain hypersensitivity in this model.

The observed behavioral manifestations, including mechanical allodynia and thermal hyperalgesia, are consistent with previous reports of neuropathic pain following peripheral nerve injury.^[4]^ The early onset and persistence of pain behaviors suggest robust and sustained nociceptive processing, implicating the involvement of central sensitization mechanisms in addition to peripheral nerve damage.^[5]^

The upregulation of pro-inflammatory cytokines (IL-1β, IL-6, and TNF-α) in the spinal cord following brachial plexus avulsion aligns with the concept of neuroinflammation as a key driver of neuropathic pain. These cytokines are known to sensitize nociceptive pathways, promote glial activation, and modulate synaptic transmission, contributing to the amplification and maintenance of pain signaling.^[8]^ Furthermore, the observed increase in anti-inflammatory cytokine IL-10 suggests a compensatory response aimed at dampening inflammation and restoring homeostasis within the spinal cord microenvironment.^[7]^

The systemic elevation of pro-inflammatory cytokines in serum reflects the systemic inflammatory response triggered by peripheral nerve injury. This systemic component may contribute to the amplification of pain signaling through bidirectional communication between the peripheral immune system and the central nervous system. The correlation between cytokine levels and behavioral parameters supports the notion that cytokine dysregulation contributes to the development and progression of neuropathic pain.^[9]^

The early peak of pro-inflammatory cytokines followed by a gradual decline suggests an initial robust inflammatory response that subsides over time. In contrast, the delayed increase in anti-inflammatory cytokines may reflect the body’s attempt to restore homeostasis and mitigate ongoing inflammation. The temporal dynamics of cytokine expression reveal distinct patterns of cytokine release, with pro-inflammatory cytokines peaking early post-surgery followed by a gradual decline, whereas anti-inflammatory cytokine levels exhibit a delayed but sustained increase. These temporal changes likely reflect the complex interplay between different immune cell populations and signaling pathways involved in the resolution of inflammation and tissue repair processes.^[7]^

## 5. Limitations and future directions

Despite the insights provided by this study, several limitations warrant consideration. First, the focus on male rats may limit the generalizability of findings to other populations, including female animals and humans. Sex differences in pain processing and immune responses are well-documented and should be explored in future studies. Additionally, the study period was relatively short-term, and long-term effects of cytokine dysregulation on neuropathic pain progression and resolution remain to be elucidated. Furthermore, the study primarily examined a subset of cytokines, and future investigations should explore broader cytokine profiles and their interactions in neuropathic pain pathophysiology.^[9]^

Future studies should explore the long-term effects of cytokine dysregulation and investigate sex-specific differences in cytokine responses to further refine therapeutic strategies.

## 6. Conclusion

This study underscores the pivotal role of cytokine-mediated neuroinflammation in the development and perpetuation of neuropathic pain subsequent to brachial plexus avulsion. The observed significant upregulation of pro-inflammatory cytokines, such as IL-6 and TNF-α, in the spinal cord aligns with the concept of neuroinflammation driving neuropathic pain. These cytokines are known to heighten nociceptive pathway sensitivity, stimulate glial cell activation, and modulate synaptic transmission, thereby intensifying and perpetuating pain signaling. Concurrently, the systemic elevation of pro-inflammatory cytokines in serum suggests a peripheral–central immune interaction that may augment pain signaling. The temporal correlation between cytokine level changes and the onset and progression of behavioral hypersensitivity further implicates cytokine dysregulation in neuropathic pain pathogenesis.

The increase in anti-inflammatory cytokines like IL-10 may represent a compensatory mechanism aiming to curb inflammation and reestablish homeostasis within the spinal cord microenvironment. However, this study primarily examined a subset of cytokines, and future research should investigate broader cytokine profiles and their interactions in neuropathic pain pathophysiology. The use of only male rats limits the generalizability of our findings, and sex differences in pain processing and immune responses should be explored in future studies. Long-term studies are also needed to delineate the persistent effects of cytokine dysregulation on chronic pain development.

In summary, our findings provide valuable insights into the mechanisms of pain hypersensitivity following brachial plexus avulsion and highlight targeting cytokine signaling pathways as a promising therapeutic approach for alleviating neuropathic pain in patients with peripheral nerve injuries. Future studies should explore the long-term effects of cytokine dysregulation and investigate sex-specific differences in cytokine responses to further refine therapeutic strategies.

## Author contributions

**Conceptualization:** Le Wang.

**Data curation:** Le Wang.

**Formal analysis:** Le Wang.

**Investigation:** Le Wang.

**Methodology:** Le Wang.

**Supervision:** Jie Lao.

**Writing – original draft:** Jie Lao.

**Writing – review & editing:** Jie Lao.
